# Lymphoma of the Uvula: Clinical, Morphological, Histopathological, and Genetic Characterization. A Nationwide Danish Study From 1980 to 2019

**DOI:** 10.3389/fsurg.2021.675279

**Published:** 2021-04-30

**Authors:** Lars Iversen, Patrick Rene Gerhard Eriksen, Simon Andreasen, Erik Clasen-Linde, Preben Homøe, Irene Wessel, Christian von Buchwald, Steffen Heegaard

**Affiliations:** ^1^Department of Ophthalmology, Rigshospitalet-Glostrup, Glostrup, Denmark; ^2^Department of Pathology, Rigshospitalet, Copenhagen, Denmark; ^3^Department of Otorhinolaryngology Head and Neck Surgery and Audiology, Rigshospitalet, Copenhagen, Denmark; ^4^Department of Otorhinolaryngology and Maxillofacial Surgery, Zealand University Hospital, Køge, Denmark

**Keywords:** lymphoma, uvula, head and neck, peripheral T-cell lymphoma, extranodal marginal zone B-cell lymphoma, cancer

## Abstract

**Background:** In the head and neck region the uvula is a rare site for extranodal lymphomas to develop. In this national study, we present six cases and provide an overview of the current literature, characterizing the clinical and histopathological features of lymphomas involving this location.

**Materials and Methods:** Clinical information was obtained retrospectively from patient records in a nationwide Danish study covering from 1980 through 2019. In order to validate the diagnoses, uvular tissue specimens were examined histologically and immunohistochemically and if relevant for subtyping, cytogenetic rearrangements were investigated.

**Results:** We present six cases of lymphomas involving the uvula, of which four of the cases were diagnosed with a B-cell lymphoma (two diffuse large B-cell lymphomas, one extranodal marginal zone B-cell lymphoma and one Mantle cell lymphoma), while two were diagnosed with a T-cell lymphoma (one peripheral T-cell lymphoma and one natural killer/T-cell lymphoma). Presenting symptoms included swelling, pain and ulceration of the uvula. Treatment was comprised of radiotherapy and/or chemotherapy, with T-cell lymphomas showing a poorer outcome than B-cell lymphomas.

**Conclusion:** Lymphoma of the uvula is rare, with few case reports being reported in the literature. The most frequent histological subtypes reported are extranodal marginal zone B-cell lymphoma and peripheral T-cell lymphoma. When encountering a swollen, painful and/or ulcerated uvula, the clinician should always consider malignancy as a possible cause. Lymphoma of the uvula is a possible diagnosis and if this is the case, there is a high risk of disseminated disease at the time of diagnosis.

## Introduction

Malignant lymphomas are a heterogeneous group of malignancies characterized by neoplastic proliferation of lymphoid cells. Several different subtypes are recognized by the World Health Organization Classification based on their distinctive histopathological and genetic features (1). Lymphomas often arise from lymph nodes but can also present as an extranodal disease. In 25–30% of cases, non-Hodgkin lymphomas (NHL) arising from the head and neck region are of extranodal origin (2). Furthermore, lymphoma is the third most common malignancy in the head and neck region followed by squamous cell carcinoma and adenocarcinoma (3). In descending order of frequency, the most common site for extranodal lymphoma is the ocular adnexa, sinonasal region, salivary glands, oral cavity and the larynx. Due to its richness of lymphoid tissue, the Waldeyer's tonsillar ring is also a common location for lymphomas to arise but in recent literature this location is considered a nodal site. The anatomical location of the primary presentation and dissemination of extranodal lymphomas are highly dependent on subtype. Therefore, lymphoma subtypes can vary substantially according to the location in the head and neck region. Extranodal marginal zone B-cell lymphoma (EMZL) is the most frequent lymphoma subtype seen in the ocular adnexa, while diffuse large B-cell lymphoma (DLBCL) and Natural-killer/T-cell lymphoma (NKTCL) are the most common subtypes seen in the sinonasal region. In the salivary glands and oral cavity, DLBCL, EMZL and follicular lymphoma (FL) are the most frequent subtypes (2).

Treatment of lymphomas include chemotherapy and/or radiation therapy (RT) depending on the subtype, location, extension and dissemination of the disease, while the histological diagnosis is procured surgically. Several factors affect the prognosis including histological subtype, stage at the time of diagnosis together with the age and performance status of the patient. In the head and neck region, indolent lymphomas such as EMZL and FL often have a favorable prognosis while subtypes such as DLBCL, mantle cell lymphoma (MCL) and NKTCL have a poorer prognosis (4).

Due to the lack of lymphoid tissue, lymphomas of the uvula are exceedingly rare and thus rarely described in the literature. Therefore, little is known about the presentation, distribution of subtypes, treatment and prognosis of this specific group of patients. Here, we present six new cases and characterize their clinical, morphological, histopathological and cytogenetic profile and provide an overview of the literature.

## Materials and Methods

The Danish National Pathology data bank (Patobank), which contains data from all pathoanatomical examinations performed in Denmark, was screened for all cases of lymphoma of the uvula from 1980 through 2019. The search resulted in 13 alleged cases of uvular lymphomas. Seven cases did not fulfill the inclusion criteria of a histologically verified lymphoma located to the uvula. Subsequently, six uvular specimens were requisitioned from the respective pathology departments. The number of adult inhabitants in Denmark is 4.7 million people. The following patient characteristics were obtained from patient records: age, sex, clinical presentation, clinical work-up including diagnostic imaging, stage, subtype, treatment and outcome. If possible, staging was performed using the Cotswolds-modified Ann Arbor classification and the modified American Joint Committee on Cancer (AJCC) Lugano classification. Primary lymphoma of the uvula is defined as no systemic involvement or involvement of other organs at the time of work-up and no prior history of lymphoma. Secondary lymphoma is defined as concurrent systemic disease or involvement of other organs at the time of work-up and/or prior history of lymphoma.

### Validation of Diagnoses

Formalin-fixed and paraffin-embedded (FFPE) tissue was cut and stained with hematoxylin and eosin (HE). Immunohistochemistry was performed on a Ventana Benchmark Ultra platform (Ventana Medical Systems, Tucson, AZ, USA) as previously described (5). If relevant for the suspected lymphoma subtype the following antibodies were applied: CD2, CD3, CD4, CD5, CD7, CD8, CD10, CD20, CD21, CD23, CD30, CD56, CD79a, LMP, MUM1, CXCL13, TIA1, BCL2, BCL6, PD1, cMYC, PAX5, Cyclin D1, and SOX11. Examination of the slides and validation of the diagnoses were performed by a hematopathologist.

Fluorescence *in situ* hybridization (FISH) was performed in all cases of DLBCL using the break-apart probes for MYC. If this proved positive, BCL2 and BCL6 would also be analyzed, taking into account the diagnosis of high-grade B-cell lymphoma with MYC and BCL2 and/or BCL6 rearrangements. Rearrangement analysis was performed according to the manufacturer's protocol using the HYBrite platform (Abbott Molecular). After hybridization, nuclei were counterstained with DAPI II (ZytoVision). One hundred nuclei were counted, and only nuclei where the entire nuclear membrane could be visualized were scored. Cut-off value was defined as 10%.

### Ethics

The study was approved by the Local Scientific Ethics Committee (Journal No. H-16023080) and the Danish Data Protection Agency (Journal No. P-2020-588).

## Results

A total of six cases diagnosed with lymphoma involving the uvula were included in this national study. The median age at diagnosis was 78 years ranging from 68 to 94 years with an equal male to female ratio. Four of the cases were diagnosed with a B-cell lymphoma and two of the cases were diagnosed with a T-cell lymphoma.

### Clinical Presentations

The presenting symptoms from the uvular lesions included swelling, pain, and ulceration (Table 1). In addition, case 1 presented with B-symptoms such as prolonged fever, night sweats, fatigue and a weight loss of 6 kg over 6 months. In two of the cases (case 3 and 4) the uvular process was a local extension from a tonsillar lymphoma.

**Table 1:** Demographic, clinical presentation, subtype, possible dissemination, stage, imaging, treatment and outcome of all previously published, and present uvular Lymphomas.

**Study**	**Years/sex**	**Clinical presentation**	**First presenting symptom**	**Lymphoma subtype**	**P/S/LS**	**Extra-uvular tissue involvement**	**AA/AJCC**	**Staging imaging**	**Treatment**	**Clinical course**
**B-cell lymphomas**										
Okabe et al. (6)	30/F	Swelling	Yes	EMZL	S	Breast	IV/IV	CT	CHOP	CR, NA
Walker and Heffelfinger (7)	55/F	Swelling	Yes	EMZL	S	Abdominal subcutaneous tissue	IV/IV	MRI, FBCT	Surgery	AWD, 1 yr
Present case 1	68/M	Swelling	Yes	EMZL	S	Multiple lymph nodes, kidney	IVB/IV	FBCT, MRI	R-CHOP + IT-MTX + RT + R-mt	CR, 10 yrs
Present case 2	87/F	Swelling, granulated, painful, firm	Yes	DLBCL	P	None	IA/IE	HNCT	RT	CR, 11 mo
Present case 3^**^	77/M	Ulceration, painful	Yes	DLBCL	LS	Tonsil, palatine arch, cervical lymph node	IIA/IIE	HNMRI, PET-CT	R-CHOP + RT	CR, 45 mo
Present case 4^**^	94/F	Tonsillar swelling with uvular displacement	Yes	MCL	LS	Tonsil	NA	NA	Surgery	DOC, 6 days^†^
Kusunoki et al. (8)	80/F	Swelling, elastic hard	Yes	FL	P	None	IA/IE	MRI, PET-CT	Surgery + RT	CR, 3 yrs
Singh and Singh (9)	70/F	Redness, swelling	No	NHL	S	GI-tract, extra orbital	IV/IV	NA	Surgery	NA
**T-cell lymphomas**										
Lai et al. (10)	54/F	Swelling	Yes	PTCL	S	Cervical lymph nodes	II/IIE	HNCT, FBPET	CHOP	DOC, NA
Binesh (11)	54/M	Swelling, painful, hard	Yes	PTCL	P	None	I/IE	Chest CT, abdominal US	Surgery + CHOEP + RT	CR, 15 mo
Present case 5	68/M	Firm swelling	No	PTCL	S	CNS	IVA/IV	PET-CT, MRI	Surgery + CNS-Bonn + RT	DOD, 5 mo
Azad et al. (12)	23/M	Ulceration, painful	Yes	NKTCL	LS	Soft palate	I/IE	HNCT, US, chest X-ray	CHOP	DOC, 3 mo
Present case 6	79/F	Hard, painful, ulceration	No	NKTCL	S	Nasal cavity, right auricula, Arytenoid	IVA/IV	FBCT	ACVDL^*^	DOD, 31 mo
Gomez-de la Fuente et al. (13)	45/F	NA	No	MF	S	Skin, tongue, oropharynx	IVB^‡^	Chest X-ray & -CT	CS + MTX + etoposide	D, 10 yrs
Gomez-de la Fuente et al. (13)	66/F	Swelling, painful	No	MF	S	Skin	IVB^‡^	CT	Surgery + CHOP	CR, 9 yrs

*AA, Ann Arbor; ACVDL, Adriamycin, Cyclophosphamide, Velcade, Dexamethasone, and Lenalidomide; AJCC, American Joint Committee on Cancer; AWD, Alive with disease; CNS-Bonn, MTX, Cytarabine, Dexamethasone, Vinca-alkaloids, Ifosfamide, and Cyclophosphamide incl. intraventricular MTX, Prednisolone and Cytarabine; CR, Complete response; CS, Corticosteroid; CT, Computed tomography; D, Dead; DOC, Dead of other causes; DOD, Dead of disease; EMZL, Extranodal marginal zone B-cell lymphoma; F, Female; FB, Full body; FL, Follicular lymphoma; FV, Follicular variant; HN, Head neck; IT, Intrathecal; LS, Local spread; M, Male; MF, Mycosis fungoides; mo, months; MRI, Magnetic resonance imaging; MTX, Methotrexate; NA, Not available; NHL, Non-Hodgkin lymphoma; NKTCL, Natural killer/T-cell lymphoma; P, Primary; PET, Positron emission tomography; PTCL, Peripheral T-cell lymphoma; (R)-CHO(E)P, (Rituximab), Cyclophosphamide, Hydroxydaunorubicin, Oncovin, (Etoposide), and Prednisolone; R-mt, Rituximab maintenance therapy; RT, Radiotherapy; S, Secondary; US, Ultrasound, yrs, years*.

* *Previously treated with R-CHOP, IT-MTX, RT and Methyl-GAG, Ifosfamide, MTX, and etoposide (MIME).*

** *Local spread from a tonsillar tumor*.

† *Died 6 days after surgery*.

‡ *staging for mycosis fungoides*.

### Clinical, Histopathological, Immunohistochemical, and Cytogenetic Findings

For staging purposes, further investigations included bone marrow examination and diagnostic imaging. None of the cases examined showed involvement of the bone marrow. Tissue specimens were obtained from the lesions by core needle biopsy or surgical excision with subsequent histological examination confirming the diagnosis of a lymphoma of the uvula.

#### B-Cell Lymphomas

##### Extranodal Marginal Zone B-Cell Lymphoma (Case 1)

A 68-year-old male presented with swelling of the uvula. Full body computed tomography (CT) and magnetic resonance imaging (MRI) showed enlarged mediastinal lymph nodes and a 24 mm tumor in close relation to the pericardia. Enlarged lymph nodes were also found in the retrocrural and retroperitoneal spaces and bilaterally along the iliac vessels. In addition, a 135 × 95 mm bulk tumor was found in the abdomen invading the right kidney. Thus, the lymphoma was Ann Arbor/AJCC stage IVB/IV.

**Figure 1:**
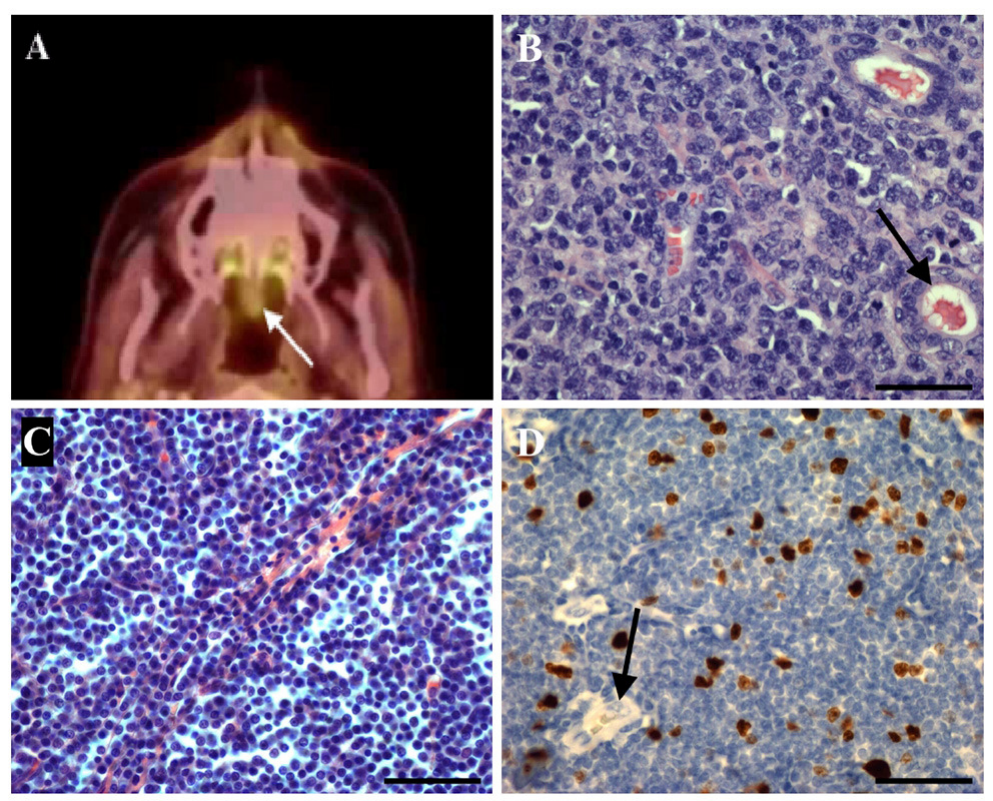
**(A)** A 68-year-old male presented with a firm swelling of the uvula and was subsequently diagnosed with a peripheral T-cell lymphoma (PTCL). Positron emission tomography-computed tomography (PET-CT) showed increased fluorodeoxyglucose (FDG)-uptake in the uvula (white arrow). **(B)** Histological image of the uvular PTCL shown in **(A)**. The uvular tissue is infiltrated by large tumor cells with irregular and vesicular nuclei. Black arrow: duct from a palatine gland (HE). **(C)** A 68-year-old male presented with swelling of the uvula and B-symptoms, including prolonged fever, night sweats, fatigue and weight loss, and was subsequently diagnosed with an extranodal marginal zone B-cell lymphoma (EMZL). The tumor tissue is characterized by small tumor cells with slightly irregular nuclei and moderately dispersed chromatin (HE). **(D)** Immunohistochemistry of the EMZL shown in **(C)**. Staining of the proliferation marker Ki-67 in the cells' nuclei shows a proliferation index of 5%. Black arrow: duct from a palatine gland. Scale bar = 50 μm.

*Histopathology* The tumor tissue consisted of small mature looking lymphoid cells with plasmacytic differentiation (Figure 1C). Immunohistochemistry showed positive reaction for CD20 and BCL2, while there was negative reaction for CD5, CD10, CD23, and cyclin D1. The proliferation marker Ki-67 showed a proliferation index of 5% (Figure 1D).

##### Diffuse Large B-Cell Lymphoma (Case 2 and 3)

An 87-year-old female presented with a swollen, firm and painful uvula. Due to old age, the patient was not a candidate for chemotherapy, thus bone marrow examination was omitted and only a CT scan of the head and neck was performed, showing no other involvement than the uvula. The lymphoma was accordingly staged as Ann Arbor/AJCC stage IA/IE.

A 77-year-old male presented with a painful, ulcerated uvula. Positron emission tomography-computed tomography (PET-CT) was performed showing increased fluorodeoxyglucose (FDG)-uptake in the right tonsil, palatine arch and uvula along with a cervical lymph node on the right side of the neck. Thus, the lymphoma was staged as Ann Arbor/AJCC stage IIA/IIE.

*Histopathology* Both tumors were dominated by tumor cells with large, pleomorphic, vesicular nuclei with distinct nucleoli. Immunohistochemistry showed positive reaction for CD20, CD79a, and BCL2. In addition, case 3 showed positive reaction for CD10, MUM, and partially for BCL6. For both cases, there was a proliferation index of 80–90% and no MYC rearrangement.

##### Mantle Cell Lymphoma (Case 4)

A 94-year-old female presented with a tonsillar swelling displacing the uvula. The patient died 6 days after surgery and was therefore not staged.

*Histopathology* The tumor was characterized by a diffuse proliferation of small homogenous lymphoid cells with pleomorphic nuclei, but without distinct nucleoli. Immunohistochemistry showed positive reaction for CD5, CD10, CD20, BCL2, PAX5, Cyclin D1, and SOX11. Furthermore, the tumor cells showed lambda light chain restriction and a proliferation index of 25%.

#### T-Cell Lymphomas

##### Peripheral T-Cell Lymphoma—Not Otherwise Specified (Case 5)

A 68-year-old male presented with a firm swelling of the uvula. PET-CT showed tumor involvement of both cerebral temporal lobes along with the uvula (Figure 1A). The lymphoma was Ann Arbor/AJCC-stage IVA/IV.

*Histopathology* The tumor was comprised of large cells with irregular and vesicular nuclei, with numerous mitotic figures (Figure 1B). Immunohistochemistry showed positive reaction for CD8, CD43, CD45, BCL2, and T cell intracellular antigen 1 (TIA1) and a partial positive reaction for CD7. Furthermore, the proliferation index was estimated to 90%. Negative reaction was found for CD2, CD3, CD4, and CD5.

##### Natural Killer/T-Cell Lymphoma, Nasal Type (Case 6)

A 79-year-old female who was originally diagnosed with an Ann Arbor stage II NKTCL located to the nasal cavity, with CT-verified bilateral involvement of cervical lymph nodes. Subsequent relapse in the right auricula, arytenoid cartilage and uvula was confirmed histologically. Further diagnostic imaging was not performed, but due to involvement of multiple non-coherent locations the lymphoma was Ann Arbor/AJCC stage IVA/IV.

*Histopathology* The tumor was characterized by diffuse infiltration of mostly small lymphoid tumor cells. There was localized necrosis along with destruction and fibrinoid changes to the blood vessels and immunohistochemistry showed positive reaction for CD2, CD3, CD4, CD5, CD8, CD43, and TIA1. Variable positive reaction was shown for CD7, CD30, CD56, and Epstein-Barr virus-encoded latent membrane protein-1 (LMP1). This is in accordance with the diagnosis of NKTCL, taking the patient's clinical history into consideration.

### Treatment and Outcome

All B-cell lymphomas received RT as part of the treatment regimen except for case 4 (MCL), who died 6 days after surgical excision of the uvular and tonsillar tumor and did not receive any treatment. For cases that were stage II or higher [case 1 (EMZL) and 3 (DLBCL)], the treatment regimen also included various chemotherapy regimens (Table 1). All B-cell lymphomas showed complete response to the treatment with no verified relapses after 10 years, 11 months and 45 months [case 1 (EMZL), 2 (DLBCL), and 3 (DLBCL), respectively] (Table 1).

The cases diagnosed with T-cell lymphoma were treated with different chemotherapy regimens. Due to involvement of the CNS, case 5 (PTCL) received intensive chemotherapy (Bonn protocol) (Table 1). The disease progressed during treatment and the patient was switched to palliative RT. Case 6 (NKTCL) was diagnosed with relapsed disease involving the uvula and was treated with chemotherapy. The patient had previously received multiple intensive chemotherapy regimens (Table 1). Case 5 (PTCL) died of the disease 5 months after the diagnosis, while case 6 (NKTCL) died of the disease 31 months after the diagnosis (Table 1).

## Discussion

Lymphoma involving the uvula is a rare malignant condition not well-described, and only a few cases have been reported in the literature. All previously published and the present cases of uvular lymphomas are presented in Table 1 and provides an overview of demographics, clinical presentation, subtype, possible dissemination, stage, imaging, treatment, and outcome (6–13). Two of the present cases (case 3 and 4) were classified as local extensions from a tonsillar lymphoma (Table 1).

Swelling of the uvula was the most common clinical presentation reported in (66.7%) of the cases, with pain (40.0%) and ulceration (20.0%) being reported as well. Possible presentations of oral cavity lymphomas have previously been reported to be localized swelling, pain and ulceration (14, 15). In comparison, other types of malignancies such as oral squamous cell carcinomas may also present as a chronic ulcer with raised margins or as a broad based exophytic mass (16). Other more common and benign causes to uvular swelling, pain and/or ulceration include viral, bacterial and fungal infections, allergic reactions, trauma and irritative conditions such as snoring. In several of the present and previously reported cases, the patients had a secondary lymphoma of the uvula with either involvement of other anatomical sites at the time of work-up or a prior history of lymphoma. Only three (20.0%) patients had a primary uvular lymphoma with no other organs or adjacent tissue involved and/or no prior history of lymphoma. Thus, when assessing a uvular lymphoma, the clinician should be aware of the possibility of systemic lymphoma with involvement elsewhere. Furthermore, it should be noticed that only few of the cases with disseminated disease had involvement of lymph nodes and those were mostly regional lymph nodes. Instead, other extranodal sites were more commonly involved. This may show a preprogrammed disposition for certain lymphomas to migrate to and colonize specific tissues (17).

Including the present and previous cases of uvular lymphomas, the most frequent histological subtypes diagnosed were EMZL and PTCL, but with a wide variation of other subtypes being diagnosed as well (Table 1). The variation of lymphoma subtypes involving the uvula stands in contrast to lymphomas arising at other locations in the head and neck region, where certain subtypes often dominate depending on anatomical site. NKTCL is seen in 70% of cases with nasal cavity lymphomas and DLBCL is seen in 50% of oral cavity and laryngeal lymphomas (2). A large US population-based study found that DLBCL was the most frequent subtype in the oropharyngeal region (18), which is not the case in the present study. In addition, the same study found only 3.6% of oropharyngeal lymphomas to be of T-cell origin with 2.2% of these being located to the pharyngeal wall. Due to the rarity of oral cavity T-cell lymphomas, publication bias in reported cases of uvular lymphomas should be considered. When the diagnosis of uvular lymphoma is established staging should preferably include bone marrow examination and full body PET-CT.

For localized indolent lymphomas, such as EMZL and FL, radiation therapy is the treatment of choice and often results in good local tumor control or complete remission. Surgery is primarily used to obtain material in order to establish a histological diagnosis or as tumor debulking. For more aggressive or advanced staged extranodal lymphomas, chemotherapy is the primary treatment modality, whereas RT may be used in specific cases as adjuvant or palliative therapy (19). Certain patients with aggressive extranodal lymphoma have an increased risk of central nervous system relapse, especially when the kidneys, adrenal glands, testicles or uterus are involved, and intrathecal administered therapy as prophylaxis should be considered for these patients (20).

In the majority of the cases, B-cell lymphomas of the uvula had a favorable outcome with complete response to the treatment in five out of seven cases. In comparison, the T-cell lymphomas had a worse outcome with only two out of seven patients showing complete response to the treatment. Two of the three cases with PTCL and both cases with NKTCL died of the disease or of other causes. In general, factors correlating to a poor prognosis in oropharyngeal lymphomas include: old age, advanced stage, B-symptoms, tumors originating from the soft palate and T-cell lymphomas (18). Especially extranodal NKTCL located outside the nasal cavity shows poor prognosis with reports of a 2-year overall survival (OS) of 34% and a 5-year OS of only 12% (21).

In conclusion, based on our national study, we present six cases of lymphoma involving the uvula along with an overview of the literature. The subtypes most frequently seen were EMZL and PTCL. Possible clinical presentations of uvular lymphomas include swelling, ulceration and pain, and thus lymphoma should be considered a differential diagnosis to other malignant lesions in this region, especially in patients with a prior history of lymphoma. Treatment should be tailored to the specific patient taking subtype and stage into consideration.

## Data Availability Statement

The original contributions presented in the study are included in the article/supplementary material, further inquiries can be directed to the corresponding author.

## Ethics Statement

Written informed consent was obtained from the individual(s) for the publication of any potentially identifiable images or data included in this article.

## Author Contributions

EC-L: validation of histological diagnoses. SA and PE: analysis of FISH. LI: first draft. All authors conception and design, critical revision for intellectual content, approval of the final version, and accountable for all aspects of the work.

## Conflict of Interest

The authors declare that the research was conducted in the absence of any commercial or financial relationships that could be construed as a potential conflict of interest.
